# Epidemiology of patients assessed for trauma by Swedish ambulance services: a retrospective registry study

**DOI:** 10.1186/s12873-023-00924-5

**Published:** 2024-01-08

**Authors:** Glenn Larsson, Christer Axelsson, Magnus Andersson Hagiwara, Johan Herlitz, Håkan Klementsson, Thomas Troëng, Carl Magnusson

**Affiliations:** 1 PreHospen-Centre for Prehospital Research, Faculty of Caring Science, Work Life and Social Welfare, University of Borås, Allegatan 1, 501 90 Borås, Sweden; 2 Department of Prehospital Emergency Care, Sahlgrenska University Hospital, Gothenburg, Sweden; 3 PICTA, Prehospital Innovation Arena, Lindholmen Science Park, Gothenburg, Sweden; 4 Register Centre South, Karlskrona, Sweden; 5 Department of Molecular and Clinical Medicine, Institute of Medicine, Sahlgrenska Academy, University of Gothenburg, Gothenburg, Sweden

**Keywords:** Trauma, Injury, Emergency medical services, Ambulance services, Patient, Severity, Mortality

## Abstract

**Background:**

There is a lack of knowledge regarding the epidemiology of severe trauma assessed by Swedish emergency medical services (EMS).

**Aim:**

To investigate the prevalence of trauma in Sweden assessed by EMS from a national perspective and describe patient demography, aetiology, trauma type, prehospital triage and clinical outcomes.

**Methods:**

Data from two national quality registries, the Swedish Ambulance Registry and the Swedish Trauma Registry (SweTrau) were collected from January 1 to December 31, 2019. Inclusion criteria were an Emergency Symptoms and Signs code equivalent to trauma in the Swedish Ambulance Registry and criteria fulfilled for SweTrau inclusion. Exclusion criteria were patients < 18 years old, those not transported to a hospital and those without a personal identification number.

**Results:**

In total, 53,120 patients with trauma were included (14% of primary EMS missions involving a personal identification number). Of those, 2,278 (4.3%) patients (median age: 45 years; 32% women) were reported in SweTrau to have severe or potentially severe trauma (penetrating: 7%, blunt: 93%). In terms of including all causes of trauma, the code for ‘trauma alert activation’ was most frequent (55%). The most frequent injury mechanism was an injury caused by a car (34%). Most (89%) cases were assigned Priority 1 (life-threatening condition) at the dispatch centre. 62% were regarded as potentially life threatening upon EMS arrival, whereas 29% were assessed as non-life-threatening. Overall, 25% of the patients had new injury severity scores > 15. 12% required invasive treatment, 11% were discharged with severe disability and the 30-day mortality rate was 3.6%.

**Conclusion:**

In this cross-sectional study, 14% of the primary EMS missions for one year were caused by trauma. However, only a small proportion of these cases are severe injuries, and the risk of severe disabilities and death appears to be limited. The most frequent aetiology of a severe trauma is injury caused by a car, and most severe traumas are blunt. Severe traumas are given the highest priority at the dispatch centre in the vast majority of cases, but nearly one-third of these cases are considered a low priority by the EMS nurse. The latter leaves room for improvement.

**Supplementary Information:**

The online version contains supplementary material available at 10.1186/s12873-023-00924-5.

## Introduction

Trauma is a significant cause of human suffering and disability, resulting in 4.4 million deaths worldwide every year [1]. The types of injury are defined as either blunt or penetrating trauma, and the rate of fatal injuries in the early phase after a severe trauma is high, with 50–60% of these injuries being related to the head, neck, thorax, abdomen and pelvis [2–4]. In Sweden, a considerable proportion (15%) of contacts with the ambulance services (AS) is caused by low- or high-energy trauma incidents [5]. The latter is the most common cause of death among younger people and of the male gender [6].

The prehospital triage of a patient’s condition and the mechanism of injury are essential aspects of the trauma care system that play a role in improving survival and decreasing hospitalisation for severe trauma patients. To streamline trauma care, national trauma alert criteria have gradually been implemented since 2016 and are used by most of the ambulances and hospital organizations [7]. Furthermore, emergency hospitals report data on patients who have sustained severe injuries in the Swedish Trauma Registry (SweTrau) for comparison and outcomes of trauma care [8]. However, there is a lack of national registration of ambulance assignments regarding occurrence and type of injury, prehospital triage and clinical outcomes. This means that knowledge regarding the scope and care results for patients who trigger a priority 1 or 2 response by ambulance, but later are found not severely injured is limited. Increased knowledge is required to improve prehospital triage.

A contributing reason for the knowledge gap in this area might be the lack of national registration and matching between registers that map injuries of varying severity.

To improve prehospital emergency care and evaluate the effect of early assessment and care measurements, a national ambulance registry, the Swedish Ambulance Registry (AmbuReg), is under development. The registry started in 2016, and since 2019, data from all AS in Sweden is reported. The present study included matched patient data from AmbuReg regarding AS clinical assessment, triage, care measurements and the more detailed records from SweTrau. Such a background information is of importance for the future planning of early trauma care in Sweden. The aim of this study was to investigate the prevalence of trauma in Sweden assessed by emergency medical services (EMS) from a national perspective and to describe patient demography, aetiology, type of trauma, prehospital triage and clinical outcomes.

## Methods

### Study design, sample and setting

This is a retrospective registry study based on data from AmbuReg and SweTrau. Patients aged 18 years of age and older, registered in AmbuReg from 1 January to 31 December 2019 with a main complaint indicative of injury/trauma, who were transported to an emergency hospital were collected. These patients were then matched against occurrence in SweTrau within the same period, which resulted in the final study population.

Sweden has 21 regions comprising 10.3 million residents, with approximately one million primary ambulance assignments/year, and approximately 75% of cases are transported to one of 68 emergency hospitals. According to Swedish legislation, all ambulances are staffed with at least one registered nurse and one emergency medical technician [9].

Most ambulance organisations, 19 of 21 regions use the Rapid Emergency Triage and Treatment System (RETTS) to assess patients’ conditions. RETTS [10] is a triage and priority model based on vital signs (level of consciousness, respiration rate, oxygen saturation, heart rate, blood pressure and body temperature) and Emergency Symptoms and Signs (ESS) codes, which concern the reasons patients call for help. RETTS comprises 133 different ESS codes covering the most common patient presentations, including medical, surgical, psychiatric, obstetric and paediatric complaints (2019 version). A final triage colour is determined by either deviating vital signs or identifying ESS for the present ailment. The EMS clinician assigns one of four colours to the patient’s injury severity: red means a life-threatening condition; orange, a potential life-threatening condition; and yellow and green, not life-threatening. Red and orange indicate direct assessment by a physician when arrival at the emergency department, whereas yellow and green mean a lower medical risk. Green is the lowest triage level with a need for an emergency department physician evaluation (Supplementary figure). In addition to ESS codes for isolated anatomical injuries or medical conditions, RETTS includes a multitrauma code– ESS 38. It is based on the national trauma alert criteria [7] and is used for patients with high risk of serious injuries (i.e. physiologic disturbance or severe/multiple injuries and mechanisms of injury) and the hospital should be alerted.

### AmbuReg

In 2016, AmbuReg started collecting national data on all primary ambulance assignments for patients aged 18 years and older. Since 2019, all ambulance organisations in Sweden have reported data to AmbuReg, and from 2020, all ages have been included. Approximately 900,000 primary ambulance assignments/year are registered in AmbuReg. In the present study, only patients with a personal identification number, information on time and date of the event and a registered ESS code that indicated the kind of injury or trauma and were transported to a hospital were included.

### SweTrau

SweTrau is a national quality register established in 2011 using the Utstein Trauma Template for Uniform Reporting of Data following Major Trauma [11]. The registry is evaluated with good validity [12]. During the study period, 46 hospitals reported to SweTrau. The new injury severity score (NISS) is used to predict mortality and describe the severity of a trauma patient’s injuries [13] and was developed from the Abbreviated Injury Scale [14]. NISS scoring ranges from 1 to 75, and a score > 15 describes a severely injured patient. The registry includes patients either admitted after trauma team activation *or* afterwards found to have a NISS > 15 at a hospital and at discharge, *and* patients who were secondarily transported from another hospital within seven days after a traumatic incident. The registry had the following exclusion criteria: 1) patients where the only traumatic injury is chronic subdural hematoma,2) patients where the trauma alarm is triggered without an underlying traumatic event and 3) patients with protected identity.

### Data collection

Data collected from AmbuReg included personal identification number, time and date of event, ambulance assignment alert times, gender, age, priority at dispatch centre, vital signs and the following ESS codes indicating injury/trauma at EMS assessment: 30, head/neck/hanging; 31, thorax/back/abdomen/pelvis; 33, arm/hand/shoulder/collarbone; 34, hip/lower extremity; 35, burn/electricity/chemicals/inhalation; 36, drowning; 37, eye; 38, multitrauma and trauma alert activation; 41, animal stings; 42, physical abuse; 86, self-injurious behaviour and finally triage assessment and triage colour. Data from nine regions were excluded because the patients’ personal identification numbers were not reported and matching against SweTrau was not possible. There was a marginal difference between the study cohort and the excluded cohort regarding the distribution of age, (median 71.0 years vs. 71.6 years), proportion of females (51.3% vs. 51.6%) and males (47.7% vs. 48.4%). Similar patterns were observed in dispatch centre prioritization, priority 1; 33.3% vs. 34.1%, and priority 2; 64.7% vs. 64.4%. EMS assessment according to the RETTS prioritization was for Red; 4.2% vs. 5.1%, Orange; 50.6% vs. 54.2%, Yellow; 26.8% vs. 26.4%, Green; 18.4% vs. 14.2%. The distribution of the most used ESS codes was, 30; 30.7% vs. 35.9%, 31; 9.8% vs. 10.3%, 33; 12.0 vs. 12.6%, 34; 30.3% vs. 30.8%, 35; 1.5% vs. 1.7% and for 38; 5.8% vs. 3.3%.

Personal identification numbers and time and dates of events were matched between the two registers. From SweTrau, the following data was collected: blunt/penetrating trauma, NISS, mechanism of injury, type of injury, length of hospital stay, preinjury physical status according to the American Society of Anesthesiologists classification (ASA) [15], initial treatment, Glasgow outcome score (GOS) at discharge from the hospital and mortality at 30 days.

### Data analysis

Outcome data are summarised by descriptive statistics. Numbers and proportions (%) are used for binary and categorical variables. For continuous variables not normally distributed, median and interquartiles are reported. For comparison of categorical variables between patients matched and not matched between AmbuReg and SweTrau, the Chi-2 test is used. IBM SPSS 27.0.0 was used for the statistical analyses [16]. Missing data were not replaced by substituted values.

## Results

### Patient characteristics

In total, 886,367 patients with a primary assignment were reported from all 21 regions in the national ambulance registry. Of these, 123,254 (14%) were registered with an injury/trauma code. Data from nine regions were excluded because the patients’ personal identification numbers were not reported (n = 70,134). Of the primary assignments from 12 regions, 53,120 cases were registered with an injury/trauma code and personal identification number and matched against SweTrau. We verified a significant difference between the matched and not matched patients for the following assessments: dispatch centre, priority 1; 89.8% vs. 32.3%, and priority 2; 10.2% vs. 65.8% (*p* = 0.0001). EMS assessment and RETTS prioritization; Red; 12.9% vs. 2.1%, Orange; 48.8% vs. 45.1%, Yellow; 10.3% vs. 24.9%, Green; 18.9% vs. 20.3% (*p* = 0.0001). The distribution of the most used ESS codes: 30; 23.3% vs. 28.3%, 31; 13.6% vs. 9.2%, 33; 2.0 vs. 10.6%, 34; 4.6% vs. 31.5%, 35; 1.1% vs. 1.7% and 38; 54.7% vs. 4.2% (*p* = 0.0001).

**Fig. 1:**
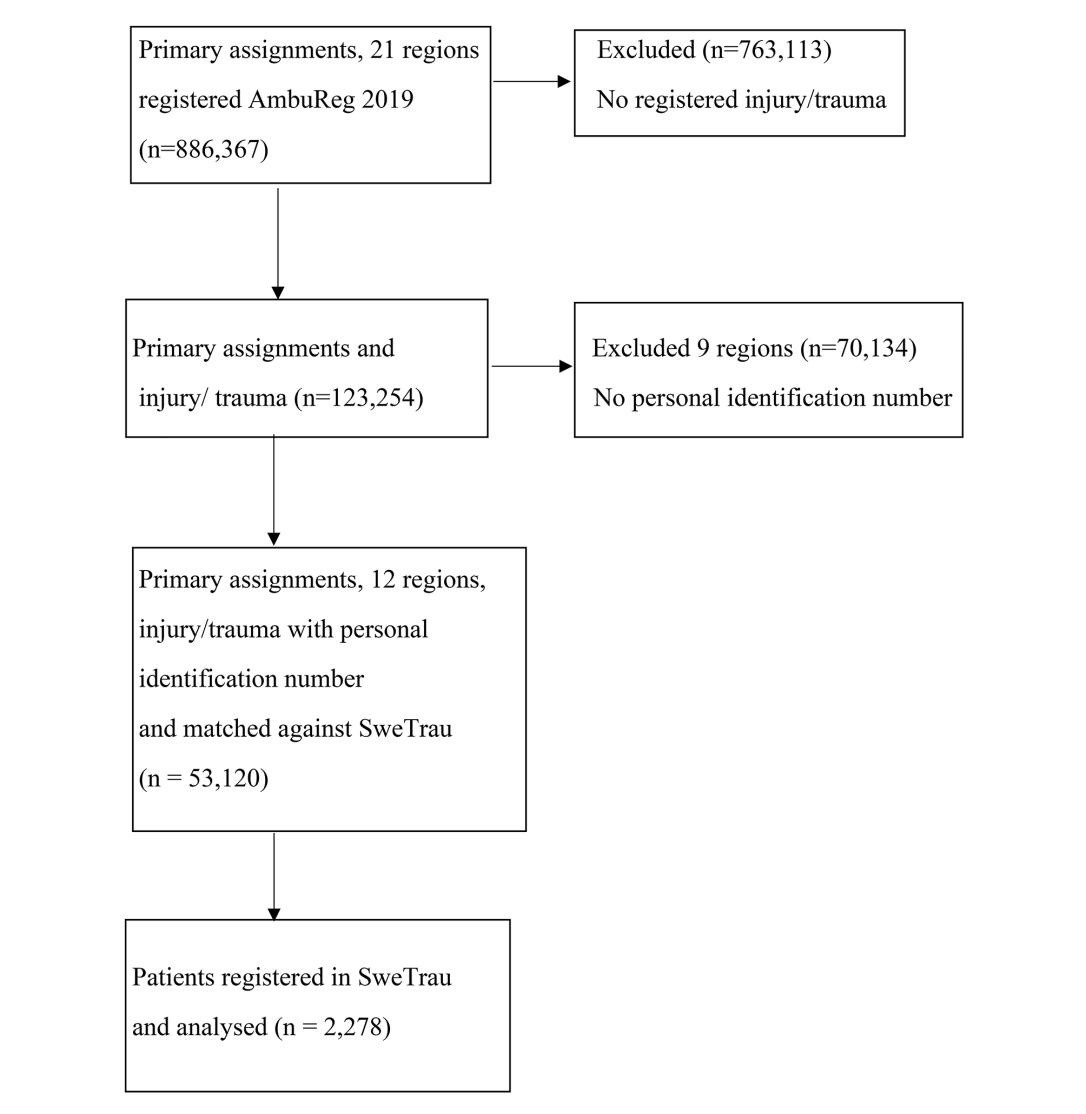
Flow of patients in the study

Among the 53,120 cases were 2,278 patients (4.3%) reported in SweTrau due to severe or potentially severe trauma (Fig. 1). This was the study population.

Of the included patients, 1,555 were males (68%) and 723 (32%) were females and the median age was 45 years (p25, 28.8 years and p75, 63.0 years).

The comorbidity preinjury rate, according to the ASA classification, shows that in ASA I, 1,262 (55%) of the patients were classified as healthy, ASA II, 606 (27%) had a mild systemic disease, ASA III, 371 (16%) had a severe comorbidity and in ASA IV, 30 (1%) had a severe and continuously life-threatening comorbidity. The ASA classification was missing in nine patients.

In 90% of the cases, the dispatch centre gave Priority 1. Upon EMS arrival, the distribution according to RETTS priority showed that 1,407 patients (62%) were assessed as life threatening or potentially life threatening, 295 red (13%) and 1,112 orange (49%). Only 667 patients (29%) were assessed as having low priority, yellow 237 (10%) and green 430 (19%). However, 26% of patients with a NISS > 15 were placed at the yellow or green triage level. The RETTS priority was missing in 204 (9%) of the cases.

On EMS arrival, 14% of patients were assessed with a Glasgow Coma Score of less than 13 and showed some degree of decreased consciousness, of which more than 50% were still able to communicate. Information on level of consciousness was missing in 31% of cases.

### ESS code and mechanism of injury

The distribution of patients according to the ESS code showed that the most frequent cause of contact was code 38:‘trauma alert activation’ 1,246 (55%), which included the major groups of multitrauma or mechanisms of injury with a potentially high severity risk that need emergency management. Other codes discovered were code 30: isolated trauma to the head and neck or hanging 531 (23%), code 31: trauma to the thorax, back or abdomen 310 (14%) and code 34: trauma to the hip and lower extremities 104 (5%), code 33: trauma upper extremity 46 (2%) and code 35: burn/inhalation/chemicals 25 (1%).

**Fig. 2:**
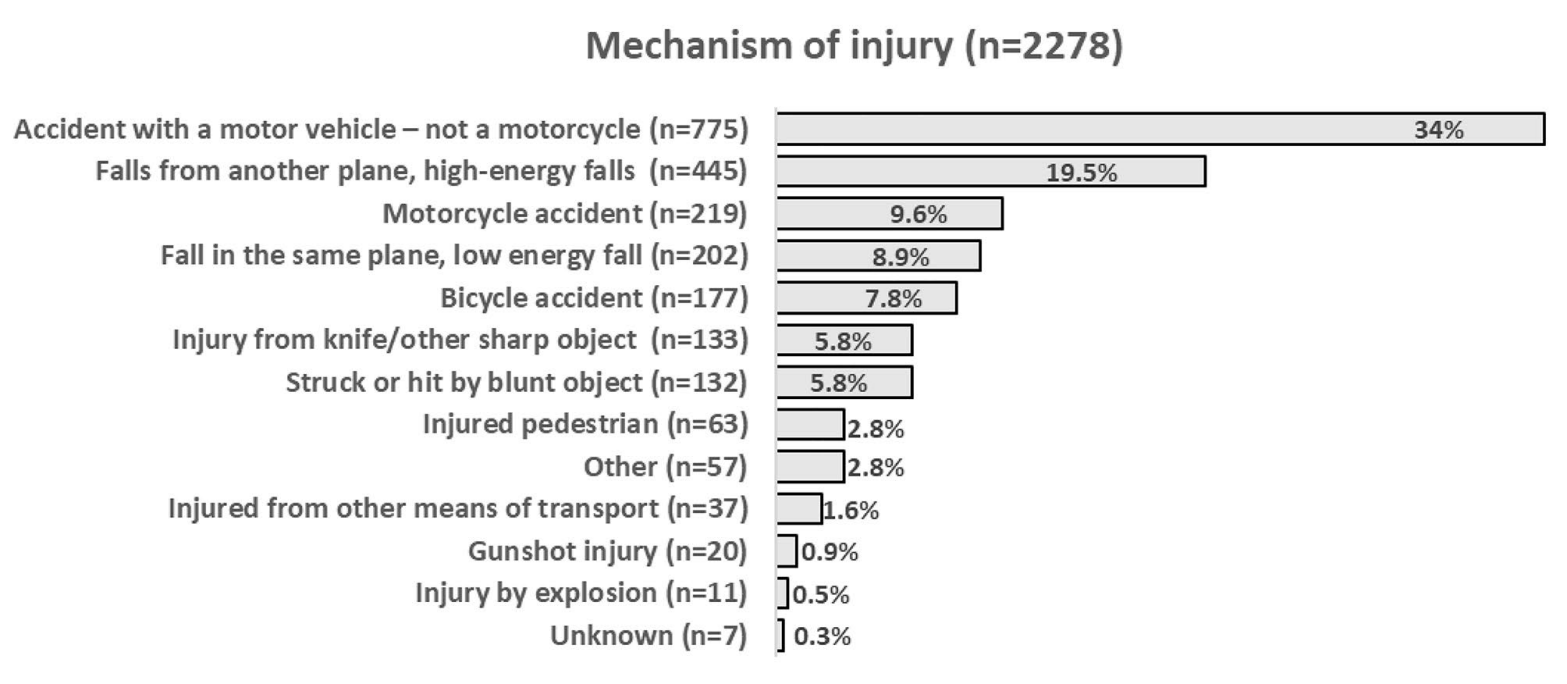
Distribution of mechanism of injury according to the Swedish Trauma Registry

The mechanism of injury showed that the most frequent cause of trauma was an injury caused by a car, 775 (34%). This was followed in order of frequency by falls from a higher level, 445 (19%), a motorcycle injury 219 (10%), falls at the same level 202 (9%) and a bicycle injury 177 (8%) (Fig. 2).

### Status on admission

According to SweTrau registration, the dominant injury type was blunt trauma (93%). A quarter of the patients (n = 572) were considered to have a NISS > 15 and three quarters (n = 1,703) had a NISS < 15. NISS score was missing in three patients. Of the 582 patients assessed with head trauma at hospital, only 2% suffered from both severe trauma and unconsciousness at the scene.

### Treatment and outcome

**Fig. 3:**
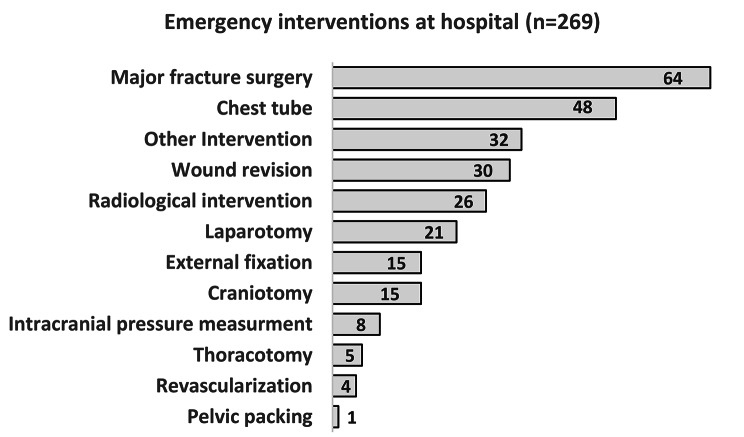
Distribution of 269 patients who required emergency interventions at hospital

In 88% of the patients, none of the defined emergency interventions at the hospital were performed (n = 2,005). 12% of the patients (n = 269) required emergency interventions for treatment and stabilisation and was performed in 85% of the patients within 12 h (median 2 h and 23 min). The most frequent treatment was major fracture surgery, followed by chest tube, other interventions, wound revision in an operation theatre, radiological intervention, laparotomy, external fracture fixation and craniotomy (Fig. 3).

**Fig. 4:**
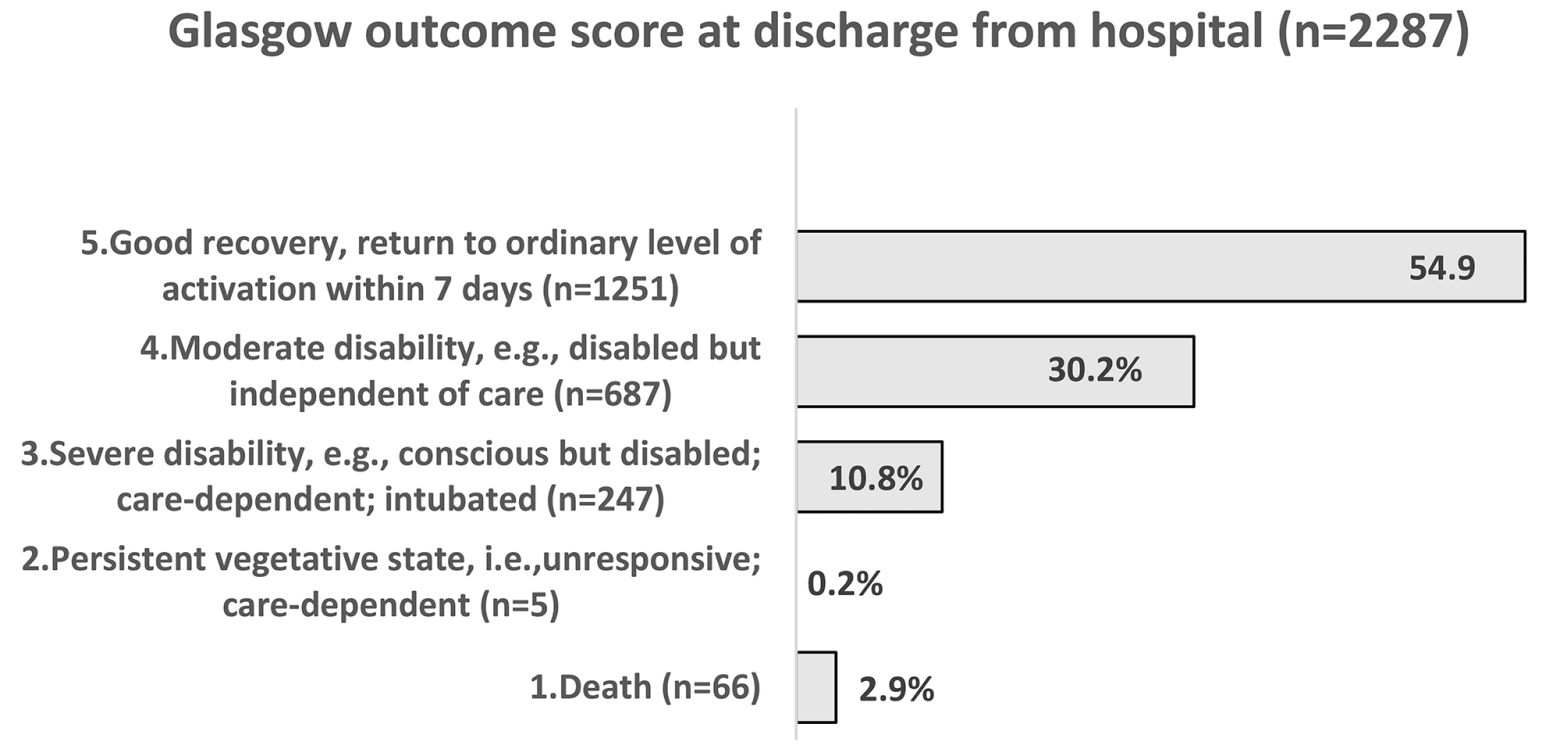
Glasgow outcome score at discharge from hospital

The median duration of hospitalisation was 2.0 days (p25, 1.0 and p75, 5.0). In terms of the Glasgow outcome score at discharge from hospital (n = 2287), 5 patients (0.2%) had a persistent vegetative condition, 66 patients died in hospital (2.9%), 247 (10.8%) percent developed a severe disabling condition, and 687 (30.2%) recovered with a nondisabling condition (i.e. they were able to live at home). Among the discharged patients 1251 (54.9%) returned within one week to the same level of function as before the trauma (Fig. 4). Information on outcome at discharge from hospital was missing for 22 patients (1%). The overall 30-day mortality rate after the trauma was 3.6% (n = 81) and 10.8% (n = 62) for patients with NISS > 15, respectively 1.1% (n = 19) with NISS < 15.

## Discussion

This study presents a nationwide description of the epidemiology of severe trauma assessed and triaged by the EMS in Sweden by combining two nationwide quality registries. The study found that 14% of the EMS population was exposed to an injury or traumatic incident. However, in 43% of the cases personal identification numbers was lacking. From the final AmbuReg population of 53,120 patients, 4.3% of these patients were registered in SweTrau with severe trauma or were exposed to a traumatic incident. This is a slightly lower incidence rate compared to other studies. A multicentre study involving Japan, Korea, Malaysia and Taiwan and including 24,365 trauma patients reported that 6.7% were severely injured [17]. However, a study from Australia reported 2.7% of acute trauma patients in the EMS [18]. The difference in incidence might depend on different safety approaches or use of protective equipment and the use of separate scales to identify severe kinematics or severely injured patients by EMS and at hospitals. Models to aid in the identification of major trauma at the scene with high accuracy are lacking. Suggestions have been made to include a combination of two physiological parameters: mechanism of injury and the patient’s comorbidity [19].

The spectrum of different ages ranged from 18 to 101 years old, with a median age of 45 years. Previous studies have reported a similar age distribution worldwide among injured patients transported by EMS for trauma care at hospitals [20, 21]. However, the incidence of trauma seems to increase in persons > 45 years old, and the highest rate is found among persons > 85 years old [18]. In agreement with previous studies, the majority (two-thirds) were men [21], and this is also true for deaths caused by injury [22]. Factors such as being a young male and having a low socioeconomic status increase the risk of being exposed to injury and of being a victim or perpetrator of serious physical violence [1].

The evaluation of comorbidity indicated that more than half of the study cohort was regarded as previously healthy according to ASA. One of the reasons for this finding may be that healthy persons are younger and more often exposed to the risk of severe trauma in comparison with elderly persons. However, elderly patients’ vulnerability to trauma has previously been described [23], and comorbidity and polypharmacy may lead to balance disorders and muscle weakness in this population, thus making them more vulnerable to trauma.

Most of the patients were given the highest priority (lights and sirens) by the dispatcher. This finding suggests a high sensitivity for an alarming situation at the dispatch centre– indeed higher than for other time-sensitive conditions, such as myocardial infarction [24] and stroke [25]. However, in agreement with many other conditions, the proportion regarded as potentially life threatening by the EMS clinician was lower (62%) in comparison with telephoned-based assessments at the dispatch centre. Telephone-based assessments are more difficult than on-scene assessments [26]. Nevertheless, a considerable proportion of patients (NISS > 15) needed comprehensive care at the hospital despite being assessed on the scene by the EMS with low acuity to be at the RETTS yellow/green triage level. Assessing the level of severity in a prehospital context is a challenge, especially among patients with moderate or no signs of severe injury (e.g. older patients with fall injuries/low energy). Further analyses are needed to clarify whether these patients can be assessed with a higher level of accuracy when related to age and mechanism of injury. A previous study described challenges in emergency care in assessing trauma patients and the need for ambulance transport to the hospital, with the possibility of rapid CT scans at the emergency room [27].

The dominant injury was blunt, and this was expected, since previous reports have shown that trauma is often caused by traffic injuries and falls [8]. However, a nationwide study from Sweden indicated an increase in penetrating injuries (mostly gunshot and stab wounds) over the past decade [28].

The ‘trauma alert activation’ code is used to prepare the receiving hospital with information to allocate a full or limited trauma team resource based on disturbance in vital parameters or specific injuries (full) or mechanism of injury alone (limited). It was difficult to get a good overview of which part of the body that was most frequently affected by the multitrauma since more than half of the patients were assigned ESS Code 38, meaning that several parts of the body could have been affected. Thus, there is room for improved coding avoiding under-triage among elderly patients suffering from falls [7].

Even though the number of traffic injuries has decreased since the beginning of the 2000s, the evaluation of the mechanism of injury showed that injuries caused by a motor vehicle was responsible for one-third of the cases. The second most frequent type of injury was falling from a higher level (high-energy trauma). Although this type of trauma has decreased in the last three years, it still represents a considerable proportion of trauma causes [8]. Furthermore, the number of bicycle injuries has increased over the last three years.

Although 25% were classified as severe trauma according to NISS, only 12% of all patients who were transported to hospital required invasive treatment. This relatively low figure indicates that severe trauma does not always translate into the need for invasive procedures. Major fracture surgery was the most frequent procedure. This was an expected finding in agreement with previous literature, which describes the important management of patients with trauma after arrival at the hospital [29].

The overall 30-day mortality rate was quite low (3.6%) in our study cohort. Candefjord et al. [21] reported a 3.4% mortality rate if patients with trauma were transported directly to a trauma centre, compared with 4.6% if they were not. This may be compared with 4.1% in an unselected patient population assessed by EMS and categorised into all ESS codes [30]. However, these patients were older (median age: 66 years) and were generally associated with a higher burden of disease. It is important to have in mind that many deaths after trauma occur before the ambulance arrives at the site of injury, and thus these victims are often not transported to the hospital, nor included in the hospital statistics.

A quarter of the patients were classified as severely injured (NISS > 15), and 12% of the patients required an emergency intervention and stabilisation upon arrival to the hospital. About 11% were discharged with a severe disabling condition, and 3% died in the hospital. Most were discharged with the same level of function as before the injury or with a moderate disabling condition. There are some uncertainties in SweTrau and the accuracy, where GOS is not comparable between a large neurosurgical clinic where patients are transferred out early compared to a regional hospital where rehabilitation takes place before discharge [14]. However, a 12-month follow-up of trauma patients demonstrated impaired physical and psychological functions and a reduced quality of life [31].

### Strengths and limitations

The major strength of this study is that AmbuReg is a nationwide registry with high representativity. However, there are some weaknesses: in a large proportion, the personal identification number was not reported from nine regions, which was necessary to match the patients between the two registries. A national consensus regarding General Data Protection Regulation (GDPR) and possibilities for all regions to send personal identification numbers to AmbuReg is important. This is an area of improvement for quality assurance and prerequisites for national follow-up and development of Swedish ambulances services. In addition, data is missing to a greater extent from AmbuReg, (level of consciousness and RETTS priority) compared to missing data from SweTrau, (ASA classification, GOS and NISS score). This may be since AmbuReg is a younger register and does not have as much experience with quality assurance of reported data. By the time of the study, some hospitals were not connected to SweTrau, and not all the connected hospitals were reporting. The coverage rate for trauma receiving hospitals was 72.6%. That might explain why only 2,278 patients were identified in the SweTrau. Furthermore, some important information is missing, such as the mode of death and the type of disabling condition. In addition, younger persons < 18 years of age were not included in the study. Further research is needed to increase the knowledge in trauma and explore outcomes of younger patients cared for by the EMS.

## Conclusion

In this one-year cohort study, 14% of the primary EMS missions were caused by trauma. However, only a small proportion of these cases are severe injuries, and the risk of severe disabilities and death appears to be limited. The most frequent aetiology of a severe trauma is injury caused by a car, and most severe traumas are blunt. Severe traumas are given the highest priority at the dispatch centre in the vast majority of cases, whereas nearly one-third of these cases are given a low priority by the EMS nurse. The latter leaves room for improvement and more detailed analyses from dispatch centre, ambulance and patient outcomes at hospital are needed to improve accuracy.

### Implications

Based on the limitations in the early assessment of trauma patients revealed in the present survey, our next study will focus on injuries and outcomes connected to ‘trauma team activation’, ‘falls from the same level’ and why a quarter of ‘low priority patients’ (yellow/green) have severe trauma. Furthermore, the development of emergency dispatch centre decisions is needed to reduce the risk of overtriage, and the use of support by artificial intelligence or cell phone video from callers can be a solution. Furthermore, we need to improve the description of the mode of death among patients who are dead on arrival of EMS as well as the mechanism behind presumed traumatic cardiac arrests on EMS arrival in order to cover all aspects of a traumatic injury.

### Electronic supplementary material

Below is the link to the electronic supplementary material.

Supplementary Material 1

## Data Availability

The datasets generated and analysed during the current study are not publicly available due to participant anonymity issues but are available from the corresponding author on reasonable request.

## Abbreviations

EMS: Emergency medical services
AmbuReg: Swedish Ambulance Registry
SweTrau: Swedish Trauma Registry
RETTS: Rapid Emergency Triage and Treatment System
ESS: Emergency Signs and Symptoms
NISS: New injury severity score
AS: Ambulance services
ASA: American Society of Anesthesiologists classification
GOS: Glasgow Outcome Score
