# Supplementary material for: Epidemiology of patients assessed for trauma by Swedish ambulance services: a retrospective registry study
Source: BMC Emerg Med. 2024 Jan 8;24:11. doi: 10.1186/s12873-023-00924-5 (PMC10775538; doi:10.1186/s12873-023-00924-5)
Supplement: Supplementary file 1 — Supplementary Material 1 [file 12873_2023_924_MOESM1_ESM.docx]

Supplementary figure. The RETTS triage process.

Vital signs (VS):

level of consciousness

respiration rate

oxygen saturation Emergency Symptoms

heart rate and signs ESS (codes)

blood pressure

body temperature

VS triage colour: ESS triage colour:

Red Red

Orange Orange

Yellow Yellow

Green Green

The highest priority

colour from VS and ESS

gives the final priority.
